# Nurse Staffing in Nursing Homes During Hurricane Irma

**DOI:** 10.1093/geroni/igab046.1103

**Published:** 2021-12-17

**Authors:** Dylan Jester, Kali Thomas, Lindsay Peterson, David Dosa, Ross Andel, Kathryn Hyer

**Affiliations:** 1 University of California San Diego, La Jolla, California, United States; 2 Brown University, Brown University/Providence, Rhode Island, United States; 3 University of South Florida, Bradenton, Florida, United States; 4 Brown University, Providence, Rhode Island, United States; 5 University of South Florida, Tampa, Florida, United States; 6 University of South Florida, University of South Florida, Florida, United States

## Abstract

Little is known about the effect of hurricanes on nurse staffing in nursing homes. Hurricane Irma made landfall on September 10th, 2017 in Florida. This study examined daily nurse staffing levels from September 3rd-24th, 2017 in 653 nursing homes; 81 facilities evacuated and 572 facilities sheltered-in-place. Data from Payroll-Based Journaling (PBJ), Certification and Survey Provider Enhanced Reports (CASPER), and Florida’s health providers’ emergency reporting system were used. Among all facilities, we found significant increases in staffing for licensed practical nurses (p=.02) and certified nursing assistants (p<.001), but not for registered nurses (p=.10) before Hurricane Irma made landfall. In comparison to facilities that sheltered-in-place, evacuating facilities increased staffing levels of all nurse types (all p<.001). From one week before landfall to two weeks after landfall, an additional estimated $2.41 million was spent on nurse staffing. Policymakers attempting to reduce the burden of natural disasters on nursing homes should reimburse staffing-related expenses.

